# The proteomics analysis of the effects of Zhishi Rhubarb soup on ischaemic stroke

**DOI:** 10.1186/s12953-021-00181-z

**Published:** 2021-11-10

**Authors:** Jing-Hua Zhang, Yue-Jia Shao, Zhen Hui, Su-Lei Wang, Chi Huang, Yang Zhao

**Affiliations:** 1 Nanjing Chinese Medicine Hospital Affiliated to Nanjing University of Traditional Chinese Medicine, 157 Da Ming Road, Nanjing City, Jiangsu Province 210022 People’s Republic of China; 2 Nanjing University of Traditional Chinese Medicine, Nanjing, 210029 Jiangsu People’s Republic of China

**Keywords:** Ischaemic stroke, Neurogenesis, Vitamin transport, Immune response, Inflammation

## Abstract

**Background:**

Stroke has always been a major threat worldwide but is most severe in China, with 2.5 million new stroke cases each year and 7.5 million stroke survivors, placing a heavy burden on the social and national health care systems. Zhishi Rhubarb Soup (ZRS) is a traditional Chinese medicine (TCM) that has been used clinically for many years in China. To explore the potential mechanism of ZRS in the treatment of stroke, liquid chromatography with mass spectrometry (LC–MS) was performed.

**Methods:**

In this study, a quantitative proteomic method with LC–MS was used to analyse the proteomic differences between MACO samples treated with ZRS and those without ZRS treatment.

**Results:**

Liquid chromatography with mass spectrometry (LC–MS) analysis led to the identification of 35,006 peptides, with 5160.0 proteins identified and 4094.0 quantified. Significantly differentially expressed proteins were identified through data analysis, and the difference was found to be more than 1.2 times (*P* < 0.05). The Gene Ontology (GO) analysis provided a summary of the dysregulated protein expression in the biological process (BP), cell component (CC), and molecular function (MF) categories. Proteins related to brain repair, including BDNF, IL-10, IL-6, and TGF-β, were found to change significantly, partially demonstrating the effectiveness of ZRS to attenuate tissue injury.

**Conclusion:**

In this study, LC–MS/MS was performed to assess the effects of ZRS on differentially expressed proteins in rats with cerebral infarction. These promising results could help to improve the understanding of the effects of drugs on stroke.

## Introduction

Stroke has always been a major threat worldwide, but it is most severe in China, with 2.5 million new stroke cases each year and 7.5 million stroke survivors [1]. The number of stroke patients in China has risen steeply, and death from stroke accounts for roughly one-third of stroke mortality worldwide [2], which also places a heavy burden on the social and national health care systems. With the ageing of populations advancing, the situation could worsen. Limited therapies are accessible to treat stroke patients, with recombinant tissue plasminogen activator (rtPA) therapy and mechanical thrombectomy currently used in the clinic [3–7]. However, only a small percentage of ischaemic stroke patients benefit from these treatments due to the narrow therapeutic time window and absolute contraindications [7].

Stroke can stimulate the growth and differentiation of endogenous neural stem cells (NSCs) in the adult hippocampus as a defence response to damage [8, 9]. Then, new-born neurons can migrate to the brain injury site and replace damaged neurons [10]. However, new-born neurons can be killed in a short time, likely through apoptosis [8, 11–13]. Anti-neuroinflammation is an important aspect of neuroprotection [10, 14]. However, the relationships between the immune response and neurogenesis have always been controversial [14, 15]. Some studies have shown that activated microglial cells slow neurogenesis and hamper brain healing [16, 17], and neuroinflammation has also been suggested to be a target for the treatment of stroke [14, 18]; however, the immune response has been shown to promote neurogenesis [19, 20], as transforming growth factor-β (TGF-β) and IL-10 are anti-inflammatory cytokines and neurotrophic mediators [21] that suppress inflammation and facilitate axonal outgrowth and angiogenesis [20]. The complex and multifaceted immune response in ischaemic stroke is a major challenge to the development of immunomodulatory therapies, and may make this development impossible.

ZRS is a type of Chinese herbal medicine, which mainly composed of *Rheum palmatum* L. (Dahuang), Magnolia henryi Dunn. (Houpu), *Citrus aurantium* L. (Zhishi) *Scutellaria*.(Huangqin), *Costus Root* (Muxiang), and *Licorice* (Gancao). Researches have shown that ZRS could regulate inflammatory factors in MCAO model rats [22]. Modified Zhishi Rhubarb Soup could improve the symptoms of paralytic intestinal obstruction after abdominal surgery [23].

In this research, LC–MS was performed to discover the mechanism underlying the effects of ZRS on stroke. Differentially expressed proteins were identified and classified, the functions and pathways in which they are enriched were determined and compared between groups. ZRS inhibited inflammation and promoted neurogenesis and damaged-brain repair. These effects are possibly related to the upregulation of vitamin transport-related proteins.

## Materials and methods

### Materials

Protein kinase inhibitor was purchased from Calbiochem; trypsin was purchased from Promega; acetonitrile and ultrapure water (H2O) were purchased from Fisher Chemical; trifluoroacetic acid, iodoacetamide, dithiothreitol, urea, and triethylammonium bicarbonate (TEAB) were purchased from Sigma; formic acid purchased from Fluka. Zhishi Rhubarb Soup (ZRS), a Chinese herbal regimen, was purchased from the Department of Pharmacy, Nanjing Chinese Medicine Hospital Affiliated to Nanjing Universityof Traditional Chinese Medicine, decocted according to the conventional method, concentrated to 2.5 g/ml and stored at 4 °C for later use.

### Ethics statement

All animal procedures and protocols were performed in accordance with The Guide for the Care and Use of Laboratory Animals (NIH publication, 85–23, revised 1996) and were reviewed and approved by the Animal Research Committee at the National Research Institute of Chinese Medicine: IACUC protocol no. P-99-11; IACUC Approval No. A-99-1. All surgeries were performed under anaesthesia, and all efforts were made to minimize suffering.

### Animal model, grouping and administration

The MCAO (middle cerebral artery occlusion) model was established using the thread bolt method [24]. The method refers to the modified Longa suture method. Briefly, the experimental animals were anaesthetized with the administration of 10% chloral hydrate (0.3 ml/100 g) into the abdominal cavity, and they were fixed on the operating table in the supine position (the rectal temperature was controlled at 37.3 ± 0.5 °C). The common carotid artery and vagus nerve were quickly exposed and separated, and the proximal end of the common carotid artery and the external carotid artery were connected. The internal carotid artery was threaded for use, and a small opening was cut at the upper end of the common carotid artery ligation, extending from the bifurcation of the common carotid artery. Premeasured fishing line was then inserted into the internal carotid artery along the common carotid artery, and the internal carotid artery was ligated when the line reached the specified length (approximately 18 mm). Tethers of different diameters were chosen according to the animal’s weight and nutrient intake, and then, the incision was sutured. After the operation, the body temperature was maintained at 37 ± 0.5 °C with an irradiation lamp, and the rectal temperature, respiration and heart rate were monitored. Before further experimentation, the animals were maintained in a cage until they awakened.

The rats were randomized into the following three groups: the con group, con-operated rats were i.g. with an equal volume of sterile saline (Group A); the vehicle group, MCAO rats i.g. with an equal volume of sterile saline after surgery and once daily (Group B); the ZRS treatment group, for MCAO rats, the effective dose was determined to be 10 g crude drug/kg body weight, and gavage was started 3 h after model establishment and once per day for 7 days (Group C). Hippocampal tissue was collected after treatment on Day 7.

### Nerve function score

After a rat was awake for 2 h, the behaviour and neurological symptoms were observed, and a score was given according to the Longa 5-level standard scoring method: 0, normal, without any neurological deficits; 1, the front paw cannot be straightened when lifted vertically; 2, leaning to the right and rotating to the right when walking; 3, the body falls to the right side while walking; and 4, not walking spontaneously or showing signs of a consciousness disorder. According to the first score, animals with no neurological deficit, 4 points, dyspnoea, early death, or subarachnoid haemorrhage found at the time of execution were discarded. Animals excluded from the group were replaced in subsequent experiments.

### Protein extraction and trypsin treatment

An appropriate amount of tissue (rat hippocampus) was weighed into a mortar precooled with liquid nitrogen, and more liquid nitrogen were added to fully grind the tissue to powder. Then, samples of each group were added to 4-fold the volume of powdered lysis buffer (8 M urea, 1% protease inhibitor, 3 μM TSA, 50 mM NAM and 2 mM EDTA) and lysed by ultrasound. The cell debris was removed after centrifugation, the supernatant was transferred to a new centrifuge tube, and the protein concentration was determined using a BCA kit.

Dithiothreitol was added to the protein supernatant to a final concentration of 5 mM and was reduced to 56 °C for 30 min. Then, iodoacetamide was added to a final concentration of 11 mM, and the supernatant was incubated for 15 min at room temperature in the dark. The urea concentration of the sample was diluted to be less than 2 M. Pancreatin was added at a mass ratio of 1:50 (pancreatin:protein), and the protein was digested overnight at 37 °C. Finally, the protein was subjected to a second enzymatic hydrolysis for 4 h after pancreatin was added at a mass ratio of 1:100 (pancreatin:protein).

### TMT labelling

The peptides digested by trypsin were desalted with Strata X C18 (Phenomenex), freeze-dried in vacuo, and then dissolved in 0.5 M TEAB and labelled according to the TMT kit operating instructions. Briefly, the labelling reagent was thawed, dissolved in acetonitrile, mixed with the peptide and incubated at room temperature for 2 h. The labelled peptide was mixed, the salt was removed, and the sample was freeze-dried under vacuum.

### HPLC fractionation

The peptides were fractionated by high-pH reverse HPLC, and the column was an Agilent 300Extend C18 (5 μm particle size, 4.6 mm inner diameter, 250 mm length). The peptides were subjected to a step gradient of 8–32% acetonitrile, pH 9, and 60 components were separated in 60 min. Then, the peptides were combined into 9 component samples, and the combined components were vacuum freeze-dried for subsequent operations.

### LC–MS analysis

The peptides were dissolved in mobile phase A for liquid chromatography (0.1% (v/v) formic acid aqueous solution) and separated using an EASY-nLC 1000 ultra-high-performance liquid-system. Mobile phase A was an aqueous solution containing 0.1% formic acid and 2% acetonitrile; mobile phase B was an aqueous solution containing 0.1% formic acid and 90% acetonitrile. The liquid gradient settings were as follows: 0–30 min, 12% ~ 26% B; 30–52 min, 26% ~ 40% B; 52–56 min, 40% ~ 80% B; 56–60 min, 80% B. The flow rate was maintained at 320 nL/min.

The peptides were separated by an ultrahigh-performance liquid system, injected into an NSI ion source for ionization and then analysed by Orbitrap Fusion Lumos mass spectrometry. The ion source voltage was set to 2.0 kV, and the peptide precursor ions and their secondary fragments were detected and analysed by high-resolution Orbitrap. The scanning range of the primary mass spectrum was set to 350–1550 m/z, and the scanning resolution was set to 60,000; the scanning range of the secondary mass spectrum was set to a fixed starting point of 100 m/z, and the secondary scanning resolution was set to 15,000. The data acquisition mode was based on the data-dependent scanning (DDA) program; that is, the first 20 peptide precursor ions with the highest signal intensity were selected to enter the HCD collision cell, and 32% fragmentation energy was used for fragmentation after the first scan. The mass spectrometry analysis was then graded. To improve the effective utilization of the mass spectrometer, the automatic gain control (AGC) was set to 5E4, the signal threshold was set to 50,000 ions/s, the maximum injection time was set to 70 ms, and the dynamic rejection time of the tandem mass spectrometry scan was set to 30 s to avoid precursor ions.

### Database search

The resulting MS/MS data were processed using the MaxQuant search engine (v.1.5.2.8). Tandem mass spectra were searched against the human UniProt database concatenated with the reverse decoy database. Trypsin/P was specified as a cleavage enzyme allowing up to 4 missing cleavages. The mass tolerance for precursor ions was set as 20 ppm in the first search and 5 ppm in the main search, and the mass tolerance for fragment ions was set as 0.02 Da. Cysteine alkylation was set as a fixed modification, and the variable modification was the oxidation of methionine, acetylation of the N-terminus of the protein, and deamidation (NQ). The quantitative method was set to TMT-6plex, and the FDR for protein identification and PSM identification was set to 1%. Carbamidomethyl on Cys was specified as a fixed modification, and acetylation modification and oxidation on Met were specified as variable modifications. The FDR was adjusted to < 1%, and the minimum score for modified peptides was set > 40.

### Western blot analysis

Twenty micrograms of protein/well was loaded onto 10% gels for separation using sodium dodecyl sulfate-polyacrylamide gel electrophoresis (SDS–PAGE). The gels were electrophoretically transferred onto polyvinylidene fluoride (PVDF) membranes (0.45 or 0.20 μm pore size; Millipore, Billerica, MA, USA). The blotted membranes were blocked with 5% nonfat dry milk in a Tris-buffered saline solution (25 mM Tris, pH 7.5, and 150 mM NaCl) containing 0.05% Tween 20 (TBST) for 2 h at room temperature, followed by incubation with the diluted primary antibody against the target protein for 4 h at room temperature. After washing for 10 min in TBST solution, the membranes were incubated with properly diluted secondary antibody conjugated with horseradish peroxidase for 2 h at room temperature. Western blots were developed using ECL chemiluminescent reagents obtained from Thermo Scientific (Waltham, MA, USA). The β-actin level was used as the loading control.

### Statistical analysis

For protein difference analysis, the ratio of the average value of all biological replicated quantitative values of each protein in the control group is regarded as a fold change (FC). A fold change in differential expression exceeding 1.2 was used as the change threshold for significant upregulation, and the FC threshold for significant downregulation was less than 1/1.2. For biological or technical replicate samples, we used principal component analysis (PCA), relative standard deviation (RSD) and Pearson’s correlation coefficient to evaluate protein quantitative repeatability.

Other data set were illustrated using the mean ± SEM and carefully checked by SPSS 20.0 statistical analysis software (SPSS, Chicago, Illinois, USA). The MWM measurement datasets were investigated by two-way analysis of variance (ANOVA). *P* < 0.05 was considered statistically significant.

## Results

### ZRS promoted brain injury amelioration and improved nerve function

**Fig. 1:**
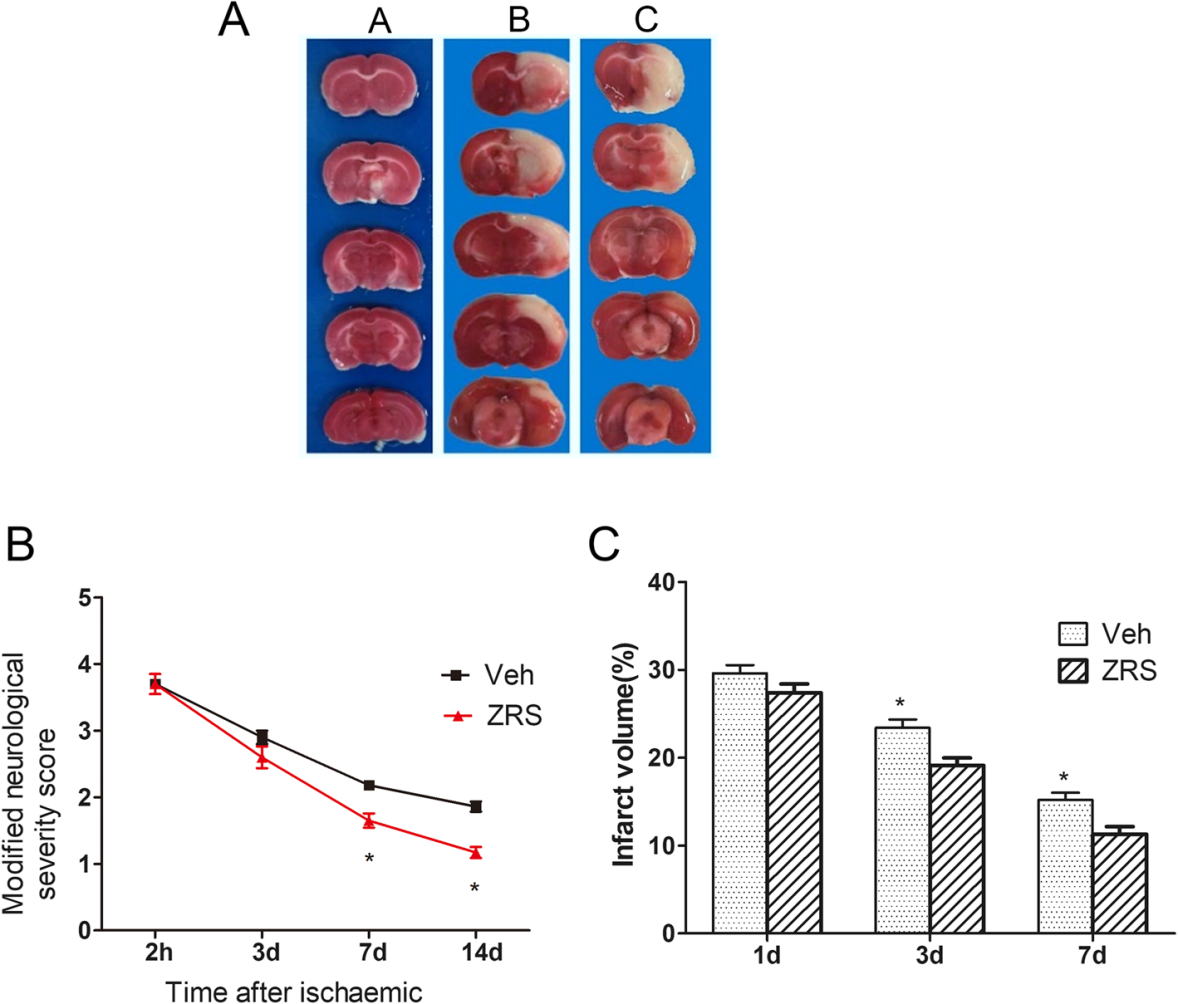
ZRS promoted brain injury amelioration and improved nerve function and behavioural performance. **A** TTC staining showing infarct volumes 7 days after surgery. Normal brain tissue appears red, and infarcted tissue appears pale grey. **B** Neurological deficit scores in the three groups were assessed by the Longa scale scoring system 2 h, 3 days, 7 days, and 14 days after reperfusion. *P < 0.05 versus Veh group, *n* = 10 per group. **C** Quantitative analysis of brain infarct volumes in vehicle- and ZRS-treated rats 1, 3, and 7 days after MCAO. *P < 0.05, **P < 0.01 versus Veh group, *n* = 6 per group

The MCAO model was established and treated with or without ZRS accordingly. On Day 7 after treatment, the behaviour of the rats was monitored to measure nerve function, and the brain was collected and subjected to TTC (triphenyl tetrazolium chloride) staining. ZRS notably decreased the infarct volume (Fig. 1A and C). In addition, the severity of nerve damage was significantly attenuated (Fig. 1B).

### Overview of the results of proteomics analysis

**Fig. 2:**
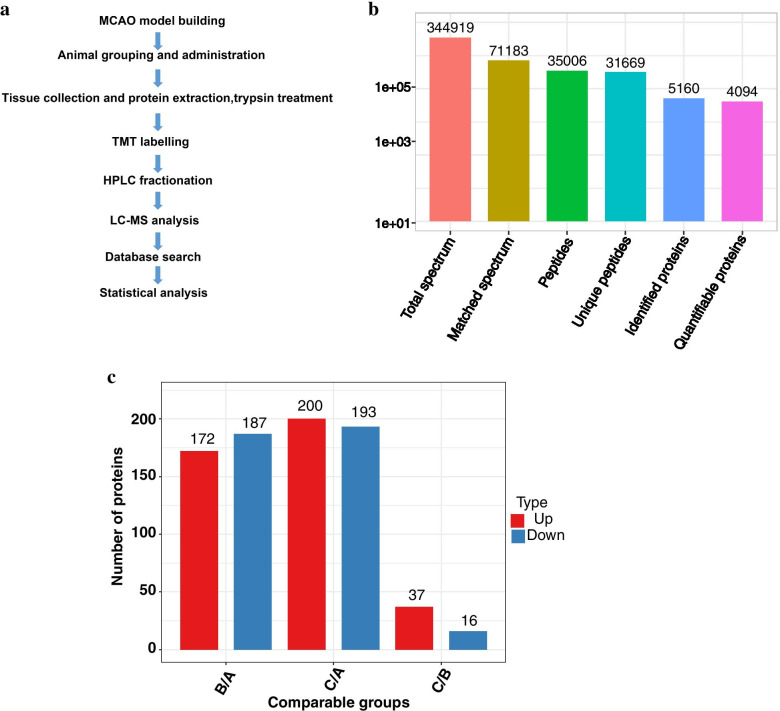
The workflow and results of proteomics analysis. **A** Workflow of the proteomics analysis. MCAO models were built, grouped, and administered to normal rats. Then, the hippocampal tissue was collected, and the proteins were extracted and digested. The proteins were later subjected to TMT labelling and HPLC fractionation and LC–MS analysis. **B** The total identified proteins in MS analysis. **C** Statistics of the differentially expressed proteins in each comparable group.

On Day 7 after treatment, the hippocampus was collected, and protein was extracted for MS analysis (Fig. 2A). The results were compared between the three groups. For each comparison group, two repeated experiments were conducted, and the shared upregulated and downregulated proteins were selected as the final differentially expressed proteins between compared groups. More attention was given to the C/B comparison group to better elucidate the mechanism of ZRS.

In the experiment, a total of 35,006 peptides were identified through spectral analysis, with 5160.0 proteins identified, of which 4094.0 could be quantified (Fig. 2B). Among the quantified proteins, 37 up- and 16 downregulated proteins were identified after the ZRS intervention (C/B); 172 up- and 187 downregulated proteins were identified after the Veh intervention (B/A); and 200 up- and 193 downregulated proteins were identified in Group C on the basis of expression differences of the proteins in Group A (C/A) (Fig. 2C).

### Analyses of gene ontology (GO), protein domains, and KEGG pathways

**Fig. 3:**
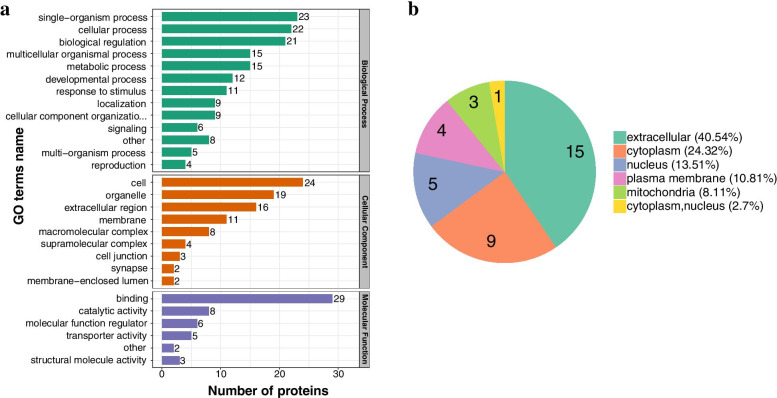
GO analysis in the C/B comparable groups. **A** Protein function analysis in terms of biological process, cellular component, and molecular function. **B** Subcellular localization of the upregulated proteins in the C/B comparable groups

To thoroughly understand the proteins identified and quantified, the functions and characteristics of the proteins were classified in terms of Gene Ontology (GO), protein domains, KEGG pathways, and the locations of subcellular structures, and then detailed annotations were made. Consistent with the mixed nature of ZRS in the C/B groups, various pharmacological effects were observed, including energy metabolism, cell proliferation and development, and general signal transduction (Fig. 3A). However, most of the upregulated proteins were located extracellularly (Fig. 3B).

**Fig. 4:**
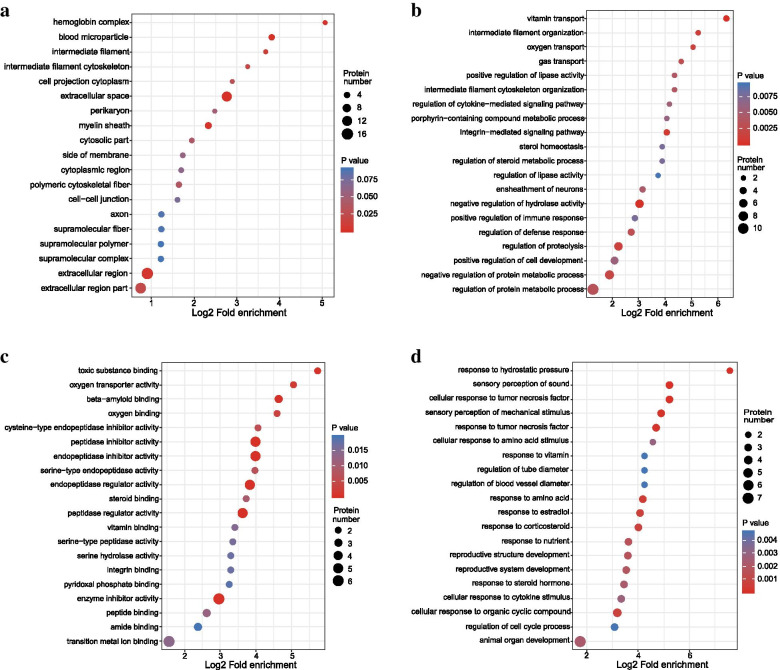
Analysis of GO functional enrichment. **A** Cellular component analysis of upregulated proteins in the C/B comparable groups. **B** Biological process analysis of upregulated proteins in the C/B comparable groups. **C** Molecular function analysis of upregulated proteins in the C/B comparable groups. **D** Biological process analysis of downregulated proteins in the C/B comparable groups

After the screening and classification of differentially expressed proteins, each comparable group was subjected to GO functional enrichment analysis and KEGG annotation. The aim was to detect whether these differentially expressed proteins exhibited significant enrichment trends in certain functional types. The highest enrichment of upregulated proteins was in the extracellular region (Fig. 4A); proteins related to vitamin transport were the most highly enriched; and proteins related to the ensheathment of neurons were also observed to be enriched to a high degree (Fig. 4B and C). In contrast to the observations in the increased-protein group, the protein response to TNF-α was found to be downregulated (Fig. 4D).

### Heat map of the cluster analysis based on GO classification

**Fig. 5:**
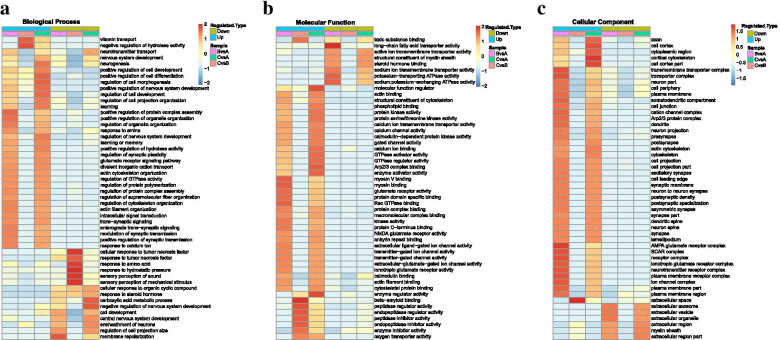
Heat map of the cluster analysis based on the GO classification. **A** Heat map analysis of the molecular process category. In the C/B groups, proteins related to neurogenesis showed a low degree of enrichment, but the proteins related to vitamin transport showed a high degree of enrichment. **B** Heat map analysis of the molecular function category. In the C/B groups, few upregulated proteins related to brain repair showed signs of enrichment, but downregulated proteins related to the response to TNF showed a high degree of enrichment. **C** Heat map analysis of the cellular component category. In the C/B groups, few upregulated proteins showed signs of enrichment, except those located in the extracellular space

To find the correlations between the functions of differentially expressed proteins in the compared groups, cluster analysis was performed. In the C/B comparison group (A), in contrast to the C/A comparison group (B), the BP analysis revealed that the degrees of protein enrichment related to neurogenesis, repair, and nervous system development were not high (Fig. 5A), but the upregulated proteins related to vitamin transport exhibited rather high degrees of enrichment (Fig. 5A). In addition, some proteins related to immunosuppression, such as TNF, showed signs of downregulation and enrichment (Fig. 5A and B). In the CC analysis, consistent with the previous protein location analysis (Fig. 3B), extracellular proteins showed a tendency towards high enrichment (Fig. 5C).

### Confirmation of differentially expressed proteins with western blot analysis

**Fig. 6:**
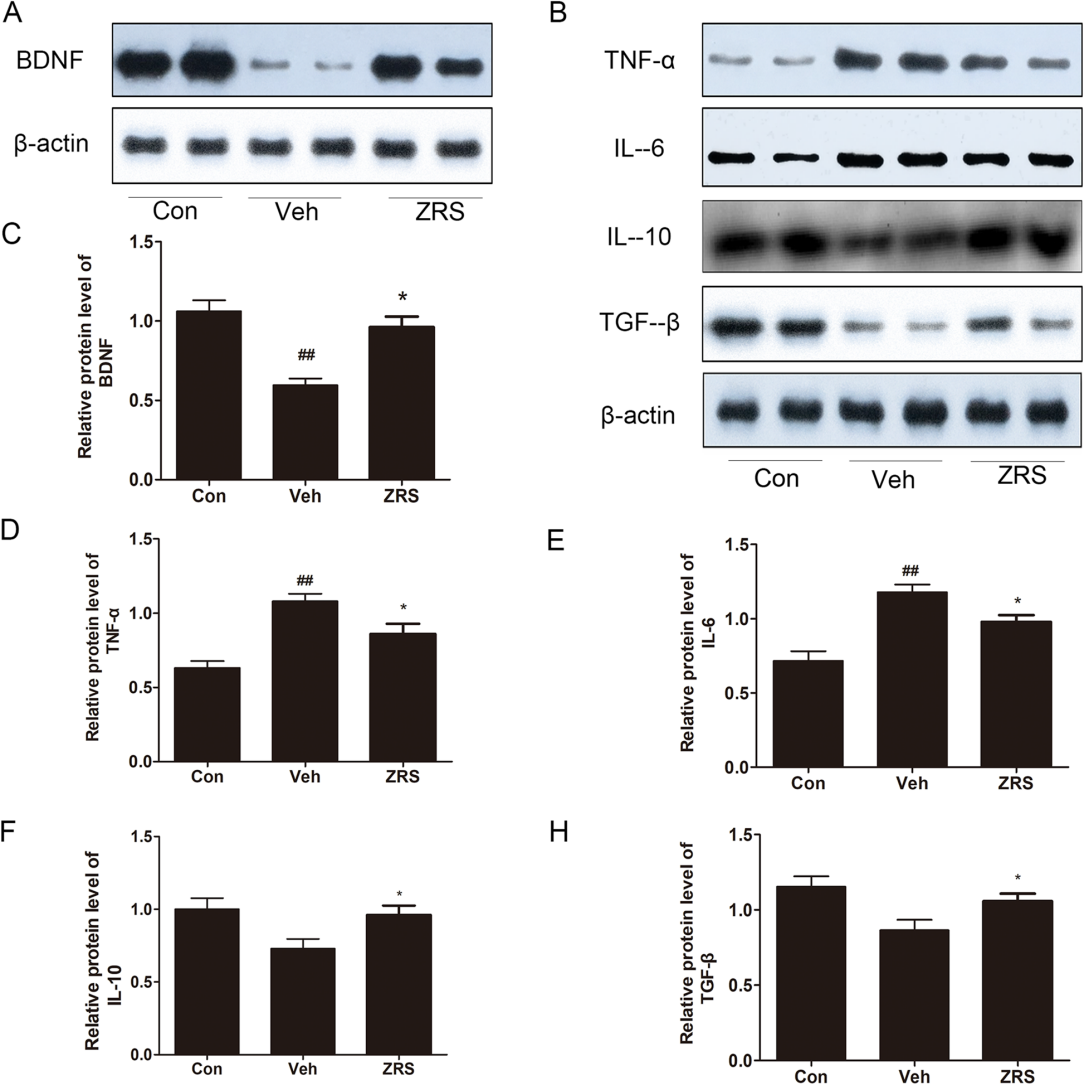
Confirmation of differentially expressed proteins. **A** BDNF expression in the ZRS group was reversed after ZRS treatment. **B** Expression levels of TNF-α, IL-6, IL-10, and TGF-β in the con, vehicle, and ZRS groups. (C-H) Quantitative analysis of BDNF, TNF-α, IL-6, IL-10 and TGF-β expression. ##P < 0.01 versus the con group; *P < 0.05 versus the Veh group. n = 6 per group

To reconfirm the observations in the MS test, western blotting was performed. BDNF is a marker of nerve regeneration [25, 26], and it was observed to be decreased in the MCAO model but the level recovered after ZRS treatment (Fig. 6A). TNF-α and IL-6 are both factors that promote inflammation [27, 28] and were upregulated in the vehicle group and downregulated in the ZRS group (Fig. 6B). IL-10 is a factor that inhibits inflammation [21, 22] and was inhibited in the treatment group (Fig. 6B). Transforming nerve growth factor (TGF), which is thought to be involved in tissue remodelling and matrix deposition [29], was shown to be elevated in the ZRS group (Fig. 6B).

## Discussion

Extensive efforts have been made to discover the pathophysiology of stroke, but without much success. Worldwide, stroke is still a major cause of mortality and disability due to the limited accessible treatment choices. Chinese herbs have previously been reported to promote neurogenesis by inhibiting inflammation [30]. In this research, we found that ZRS could ameliorate brain injury caused by stroke. In the treatment group, an improvement in brain infarction was observed after ZRS treatment compared to the MCAO group, demonstrating that ZRS promotes nerve regeneration. This outcome was confirmed by the score improvements in behaviour and neurological function. Next, a series of experiments and analyses were performed to determine the mechanisms underlying these effects. First, the differentially expressed proteins were identified, classified and subjected to Gene Oncology analysis in terms of biological process, cellular component, and molecular function. We found that the functions of proteins were highly focused on binding and transporting activities and that the proteins were primarily located in the extracellular space, which was consistent with the enrichment analysis. Extracellular proteins had the highest degrees of enrichment, including those that sheath neurons, which may indicate their involvement in the process of neurogenesis. Finally, proteins related to brain repair, including BDNF, IL-10, IL-6, and TGF-β, were found to change significantly, partly demonstrating the effects of ZRS on improving tissue injury.

In addition, vitamin transport proteins were also found to be enriched to a high degree. Notably, it had been previously found that vitamins, including vitamin D and vitamin A, can have neuroprotective effects, which can be mediated through various signalling pathways [31]. Interestingly, vitamins have also been related to immune responses [32, 33]. Therefore, interesting questions were raised regarding whether ZRS has an effect on vitamin transport and thus modulate the immune system to contribute to brain damage repair. Studies have shown that activation of vitamin receptors can affect various processes, including immune modulation, inflammation and detoxification [31]. Vitamin D hormone can stimulate transforming growth factor TGFβ-1 and interleukin 4 (IL-4) production, which in turn may suppress inflammatory T cell activity [32]. It is possible that the activation of vitamin receptors triggers the transportation of vitamins and suppresses the immune response.

The relationship between vitamins and the immune response is very complicated [34]. On the one hand, vitamins may enhance immunity [35]; on the other hand, they may also suppress immunity [36, 37]. With regard to stroke, it is very likely that, at the beginning of brain damage repair, vitamins serve to enhance immunity and eliminate infarcted nerve cells , then, change, suppressing the immune response to allow new neural stem cells to survive for a longer time. An interesting question is raised: Are the high expression and extensive enrichment of surface membrane proteins related to the transportation of vitamins? Further research is needed. In the MS data analysis, some plasma proteins, such as albumin [38] and apoa1 [39], were found to be upregulated and enriched (data not shown), and they were found to be correlated with lipid transport. Interestingly, vitamin and lipid transport processes share certain transporters [40].

In the heat map of the cluster analysis, the degrees of protein enrichment related to neurogenesis and repair and nervous system development were not high, which is probably attributable to the tissue collection timepoint. Our experiment selects brain tissue after 7 days of treatment [41].The acute phase is generally considered to last from 24 h to 1 week, but the subacute phase lasts from 1 to 3 weeks [41, 42], which can vary depending on the specific circumstances. Therefore, studies at different time points are needed to better track the mechanism by which ZHS ameliorates infarction (stroke).

Although positive results were observed, the pharmacological effects of ZRS were far too diverse due to the inherent nature of the regimen mixture; therefore, many side effects may have been revealed through the MS data analysis,but the true underlying effects have not yet been discovered, which may be related to the multi-target mechanism of traditional Chinese medicine compounds. Thus, to clarify the exact role of each component in ZRS, the isolation and purification of the botanical samples must be performed to obtain pure components.

## Conclusion

This study is the first to conduct quantitative proteomics using LC–MS/MS to identify differentially expressed proteins in stroke treated with ZRS. The results confirmed that ZRS presents a unique protein profile that indicates adaptive mechanisms in acute stroke. The therapeutic effect of ZRS may be related to its mediating inflammatory response. However, many more details and greater evidence are needed from further research to reveal the mechanism underlying the effect of ZRS on stroke.

## Data Availability

Not applicable.

## Abbreviations

MCAO: Middle cerebral artery occlusion
TNF-β: Tumour necrosis factor
tPA: Tissue plasminogen activator
VDR: Vitamin D receptor
TGF-β: Transforming growth factor-β
ZRS: Zhishi Rhubarb Soup
EDTA: Ethylenediaminetetraacetic acid
NSCS: Endogenous neural stem cells
LC–MS: Liquid chromatography mass spectrometry
NIH: National Institutes of Health
TMT: Tandem mass tags
BDNF: Brain-derived neurotrophic factor
IL-6: Interleukin-6
Apoa1: ATP-binding cassette transporter A1
